# Rice body synovitis of the shoulder joint: a case report and review of clinical management and pathology

**DOI:** 10.3389/fsurg.2026.1770970

**Published:** 2026-07-01

**Authors:** Yong Cao, Yang Li, Jun Li, Jinxin Gu, Yumei Ding, Xiaojun Ma, Jun Wan

**Affiliations:** 1 Third Clinical Medical College of Ningxia Medical University, Yinchuan, China; 2 Department of Sports Medicine, People's Hospital of Ningxia Hui Autonomous Region, Ningxia Medical University, Yinchuan City, Ningxia Hui Autonomous Region, China

**Keywords:** arthroscopy, floating lotus sign, rheumatoid arthritis, rice body synovitis, rotator cuff

## Abstract

**Background:**

Rice body synovitis is a rare subtype of synovitis, often secondary to chronic inflammatory diseases, such as rheumatoid arthritis (RA). Due to its non-specific clinical manifestations, it is prone to misdiagnosis.

**Methods:**

This report describes a 58-year-old male patient with left shoulder rice body synovitis. The patient had a 25-year history of rheumatoid arthritis and presented with a left shoulder mass for over 2 months, accompanied by pain and limited joint mobility within the previous week. Laboratory findings revealed markedly elevated rheumatoid factor levels (128.0 IU/mL) and anti-cyclic citrullinated peptide antibody levels (86.0 RU/mL), along with an elevated erythrocyte sedimentation rate (62.25 mm/h) and C-reactive protein level (15 mg/L), all of which, being above normal reference ranges, indicated active rheumatoid arthritis. An MRI showed marked capsular and bursal distension with multiple well-defined rice body-like nodules measuring approximately 0.5–0.8 cm. These nodules had low-to-intermediate signal intensity within a hyperintense effusion, producing the characteristic “floating lotus sign.” After contrast administration, the thickened synovium was enhanced, whereas the nodules showed no obvious enhancement.

**Results:**

The patient underwent arthroscopic exploration and debridement. Intraoperatively, multiple rice-grain-like bodies and proliferative synovial tissue were completely removed, followed by rotator cuff repair. Postoperative pathology revealed loose bodies composed of an amorphous necrotic core surrounded by fibrin, consistent with the pathological changes of rice body synovitis. Postoperative management included analgesic treatment, staged shoulder rehabilitation, and rheumatology follow-up for reassessment of RA activity and optimization of disease-modifying antirheumatic drug (DMARD) therapy. At the 6-month follow-up, the patient's pain had resolved, and his shoulder’s range of motion had returned to normal (180° abduction, 160° elevation). Imaging follow-up showed no recurrence.

**Conclusion:**

This single case suggests that RA-associated rice body synovitis should be considered when patients with chronic inflammatory arthritis present with persistent shoulder swelling and typical MRI findings. Arthroscopy can be diagnostically and therapeutically useful, but favorable short-term outcomes cannot be generalized from one case. Long-term follow-up and optimized RA/DMARD management remain necessary to reduce recurrence risk.

## 1 Introduction

Rice body synovitis is a relatively rare type of synovitis characterized by distinctive pathological features (1). Its defining characteristic is the presence of numerous rice-grain-like loose bodies within the joint cavity or bursa (2). These loose bodies typically measure 2–7 mm in size, exhibit an oval or spindle shape, and resemble grains of rice in appearance, hence the name (3). Rice body synovitis is not an independent disease, but rather a manifestation of various underlying conditions, frequently associated with tuberculous arthritis, rheumatoid arthritis (RA), chronic synovitis, and chronic bursitis (4). Regarding pathogenesis, it is widely recognized that rice body formation is closely linked to synovial tissue pathology (5). When the synovium undergoes inflammatory or ischemic stimulation, microinfarction occurs (6). Subsequently, these infarct sites detach and become enveloped by fibrin in the synovial fluid, gradually forming rice-grain bodies (7). Pathologically, the core of rice-grain bodies consists of amorphous eosinophilic material and collagenous tissue, tightly surrounded by a layer of fibrous tissue (8). Concurrently, the synovium exhibits villous hyperplasia accompanied by extensive lymphocytic infiltration and minor giant cell infiltration, presenting as chronic non-specific inflammation without vascular villi or granuloma formation (8).

The clinical manifestations of rice body synovitis lack specificity; patients typically present with symptoms such as joint swelling, pain, and restricted mobility, which are similar to those of other common joint disorders, leading to frequent misdiagnosis and missed diagnosis (9). Plain radiography typically reveals only periarticular soft tissue swelling and usually cannot directly visualize non-calcified rice bodies. CT scans may show dilated bursae or joint capsules with homogeneous low density, but they may not adequately depict non-calcified rice bodies. MRI, however, holds significant diagnostic value for rice body synovitis. On both T1-and T2-weighted images, rice bodies appear as isointense or hypointense signals. On T2-weighted images, they contrast sharply with the surrounding hyperintense effusion, making them easily distinguishable. After contrast enhancement, the bodies show no enhancement, while the thickened synovium and septa demonstrate enhancement (10). This feature aids in the differential diagnosis of other joint disorders (e.g., synovial osteochondromatosis or pigmented villonodular synovitis) (11).

Due to the low incidence of rice body synovitis, clinical studies have small sample sizes and an in-depth understanding is lacking, posing challenges for diagnosis and treatment (12). This report describes a case of RA-associated rice body synovitis involving the left shoulder joint. By integrating relevant literature, it provides a comprehensive analysis of the disease’s clinical features, imaging manifestations, pathological characteristics, and diagnostic/therapeutic strategies (13). Thus, this case report aims to enhance clinicians' understanding of the disease and offer a valuable reference for future diagnoses and treatments (14).

## 2 Case report

### 2.1 Clinical data

A 58-year-old male patient presented to our hospital complaining of a “left shoulder mass discovered over 2 months ago.” The patient reported incidentally noticing a lump on his left shoulder approximately 2 months prior. Initially, the mass was small and asymptomatic, so he did not pay much attention to it. Over the previous week, the mass had progressively enlarged and become painful. The pain was described as a persistent dull ache that worsened with movement, significantly impacting daily activities, prompting him to seek medical attention. The patient had a 25-year history of rheumatoid arthritis and had been treated with oral iguratimod as a conventional synthetic disease-modifying antirheumatic drug (DMARD). No biological DMARD or targeted synthetic DMARD was being used at the time of admission. He denied any history of trauma. There was also no family history of similar conditions.

Upon admission, a specialized examination revealed a soft, well-defined mass that was palpable over the left shoulder joint, with mild tenderness. No skin erythema, ulceration, or local temperature elevation was observed. Furthermore, no obvious atrophy of the deltoid, supraspinatus, or infraspinatus muscles was noted. Shoulder range of motion was restricted, with forward flexion of 110°, external rotation of 40°, abduction of 70°, and internal rotation to the L4 level.

Laboratory tests showed elevated inflammatory markers, including an erythrocyte sedimentation rate (ESR) of 62.25 mm/h and a C-reactive protein (CRP) level of 15 mg/L (Table 1). The patient’s rheumatoid factor (RF) level was 128.0 IU/mL, and his anti-cyclic citrullinated peptide (anti-CCP) antibody level was 86.0 RU/mL, consistent with seropositive rheumatoid arthritis. Other laboratory parameters, including a complete blood count and liver and kidney function tests, were within normal ranges.

**Table 1:** Preoperative laboratory tests.

Test item	Result	Normal range	Unit
Erythrocyte sedimentation rate	62.25	Male patients <15.0	mm/h
		Female patients <20.0	
C-reactive protein	15	<10.0	mg/L
Rheumatoid factor	128.0	＜20.0	IU/mL
Anti-cyclic citrullinated peptide antibody	86.0	＜5.0	RU/mL

The patient had no fever, night sweats, weight loss, wound infection, or systemic infectious symptoms. However, synovial fluid culture, mycobacterial culture, acid-fast staining, and PCR testing were not performed. Therefore, tuberculosis or other chronic infections could not be definitively excluded, and this point is acknowledged as a limitation of the study.

### 2.2 Imaging studies

#### 2.2.1 X-ray

**Figure 1:**
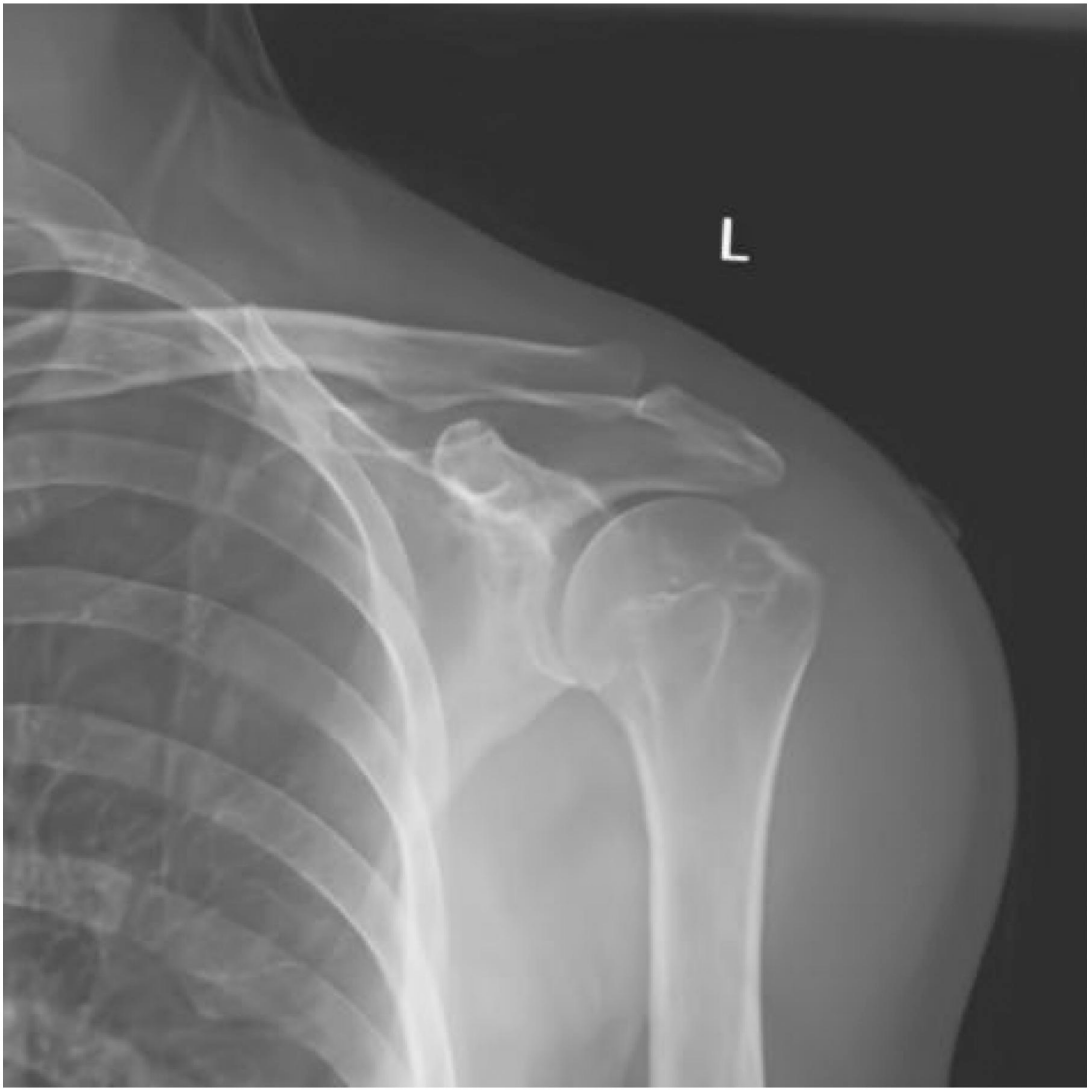
Preoperative left shoulder X-ray of the 58-year-old male patient presented in this case report.

Rice bodies are usually not visible on plain radiographs when they are not calcified, and radiographic findings are often non-specific. As shown in Figure 1, the preoperative left shoulder joint X-ray revealed no obvious abnormalities.

#### 2.2.2 MRI findings

**Figure 2:**
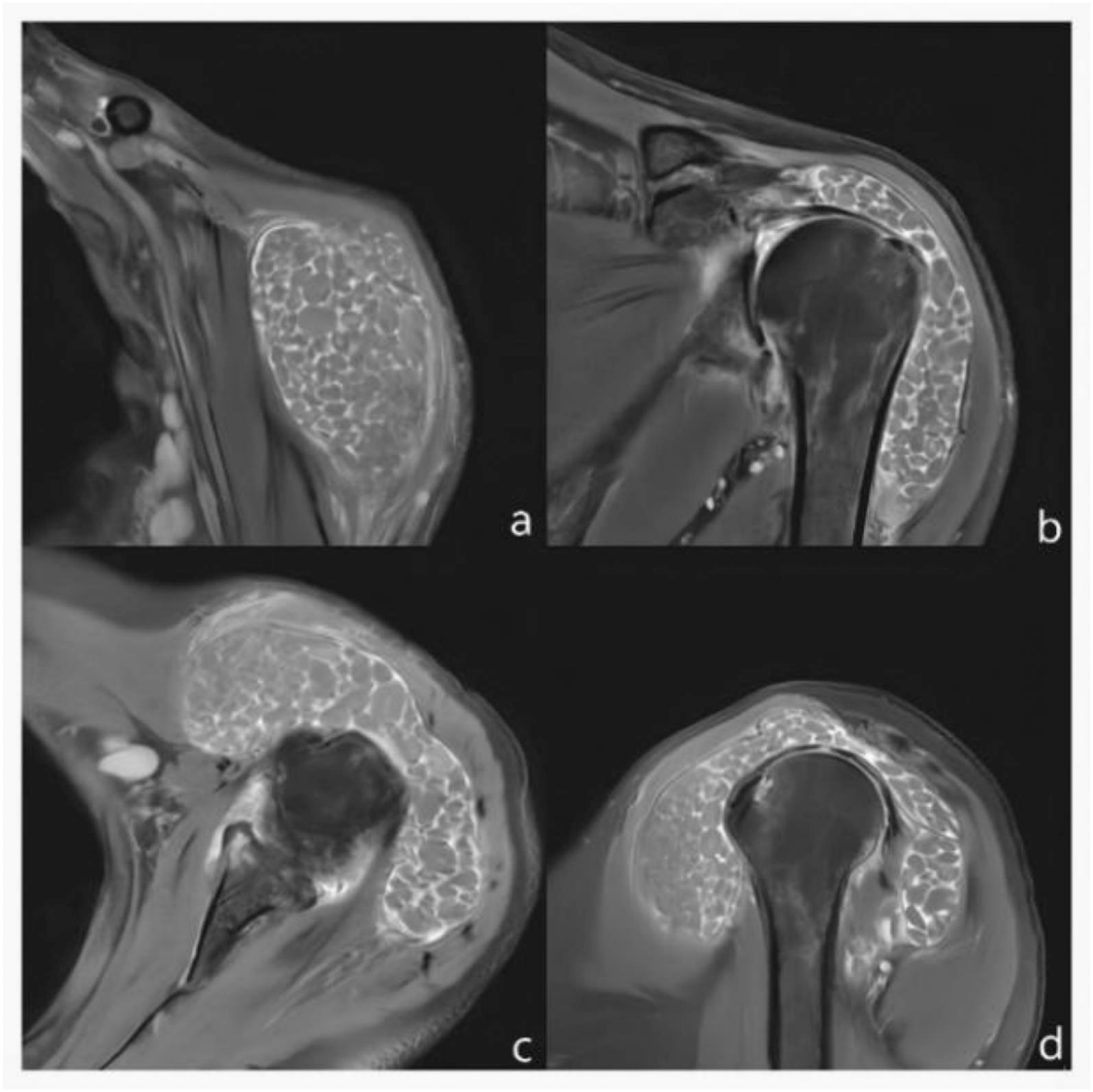
Preoperative multi-sequence MRI of the left shoulder joint in the 58-year-old male patient.

An MRI (Figure 2) showed marked distension of the left shoulder joint capsule and subacromial-subdeltoid bursa, with multiple, well-defined rice body-like nodules measuring approximately 0.5–0.8 cm. These nodules had low-to-intermediate signal intensity within a hyperintense joint effusion, producing the characteristic “floating lotus sign.” Diffuse synovial thickening was observed around the effusion, which showed enhancement after contrast administration, whereas the rice body-like nodules showed no obvious enhancement. No calcification, ossified loose bodies, or destructive bone changes were identified, which helped distinguish the lesion from synovial osteochondromatosis and infectious destructive arthritis. A concomitant supraspinatus tendon injury was suspected and confirmed intraoperatively.

### 2.3 CARE timeline

The CARE-style clinical timeline is presented in Table 2.

**Table 2:** Case report-style clinical timeline.

Time point	Clinical event	Key findings or management
25 years before admission	Diagnosis of rheumatoid arthritis	Long-term DMARD treatment; disease activity intermittently controlled
2 months before admission	Left shoulder mass first noticed	Mass was initially small and painless
1 week before admission	Pain and functional limitation developed	Mass was enlarged; shoulder motion was restricted
Admission	Clinical, laboratory, and imaging assessment	Elevated RF, anti-CCP, ESR, and CRP; MRI showed multiple floating rice-body-like nodules
Operation	Arthroscopic treatment	Loose bodies and proliferative synovium removed; bursa debrided; rotator cuff repaired
Postoperative week 1	Early recovery	Pain improved; incision healed; sling protection and rehabilitation initiated
6-month follow-up	Clinical and imaging review	No pain or mass recurrence; 180° abduction, 160° elevation; ultrasound and MRI showed no recurrent loose bodies

## 3 Surgical procedure

**Figure 3:**
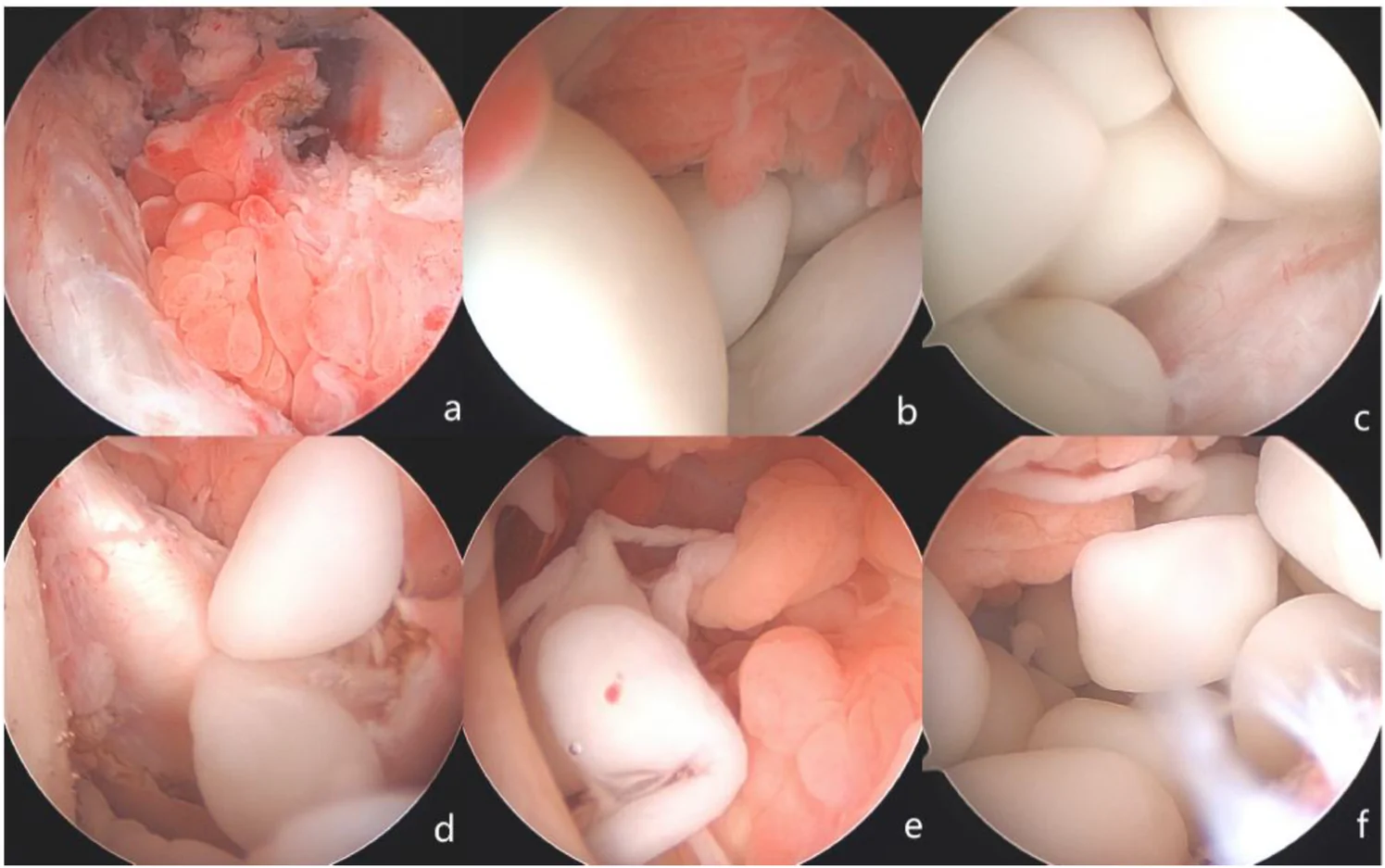
Arthroscopic findings in the left shoulder of the patient with rice body synovitis.

**Figure 4:**
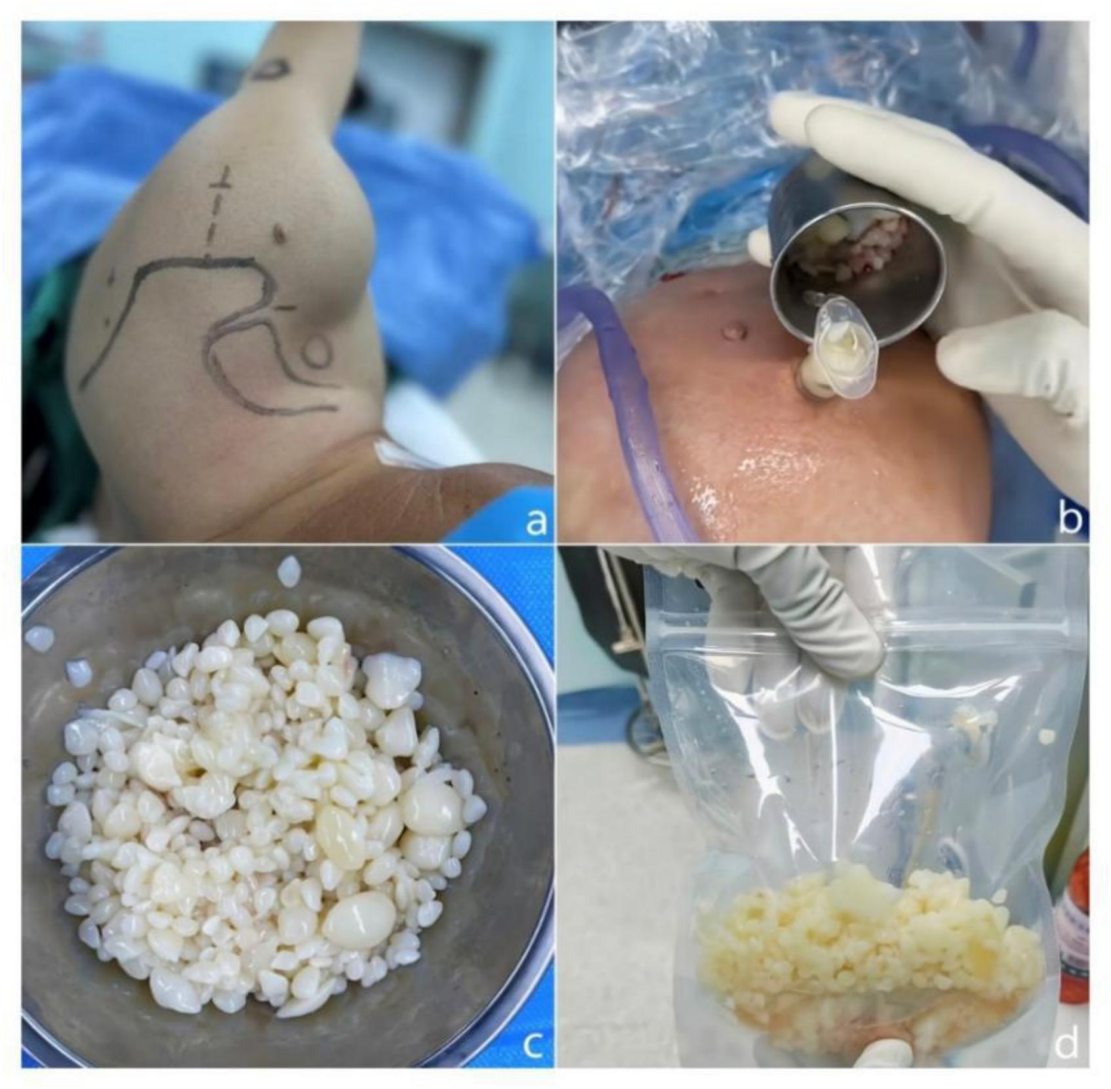
Intraoperative images of left shoulder rice body synovitis, including preoperative skin marking, arthroscopic tissue removal, rice body specimens, and specimen collection.

Under general anesthesia with controlled hypotension, the patient underwent arthroscopic exploration and debridement of the left shoulder, synovial cyst resection, long head of the biceps tendon resection, and rotator cuff repair. The patient was positioned in the left lateral decubitus position with his left shoulder suspended and tractioned (45° abduction, 20° posterior tilt, and a traction weight of 3 kg). The surgical field was routinely disinfected and draped. Exploration via posterior and anterior approaches revealed degenerative changes in the glenohumeral joint, intra-articular synovial congestion and edema, and numerous soft, grape-like masses of varying sizes exhibiting villous hyperplasia. Significant adhesions and contractures were noted in the rotator cuff space and the sulcus between the long head of the biceps tendon and the humeral tubercle. Free bodies and proliferative synovial tissue were thoroughly excised using a curette and forceps (Figures 3, 4), followed by repeated irrigation of the joint cavity and bursa. The superior joint capsule and contracted rotator cuff space were released, along with the glenohumeral ligament and anterior bundle of the inferior glenohumeral ligament. Anterior tearing of the supraspinatus muscle was observed; the subscapularis muscle remained intact with good tension. Intra-articular debridement of the masses was performed, and the long head of the biceps brachii tendon was released and transected. Access to the subacromial space via a posterior shoulder approach revealed a congested, edematous, and hypertrophic subacromial bursa. Numerous white, rice body-like loose bodies of varying sizes were also observed beneath, anterior to, and posterolateral to the acromion. Osteophytes were present at the anterolateral acromion angle, with narrowing of the subacromial space. Arthroscopic debridement and decompression of the subacromial bursa were performed. A lateral approach was used to expand the incision for drainage and to remove the masses and abnormal bursae. Intraoperatively, the anterior aspect of the supraspinatus muscle was found to be thin and torn. The footprint area where the torn supraspinatus muscle attaches was debrided and freshened. A 4.5 mm suture anchor was placed at the cartilage-bone junction to repair the torn supraspinatus tendon. Intraoperatively, the repaired supraspinatus muscle completely covered the footprint area. The joint cavity was thoroughly irrigated, and the incision was sutured. Postoperatively, the affected shoulder, neck, and forearm were immobilized with a sling for auxiliary fixation.

**Figure 5:**
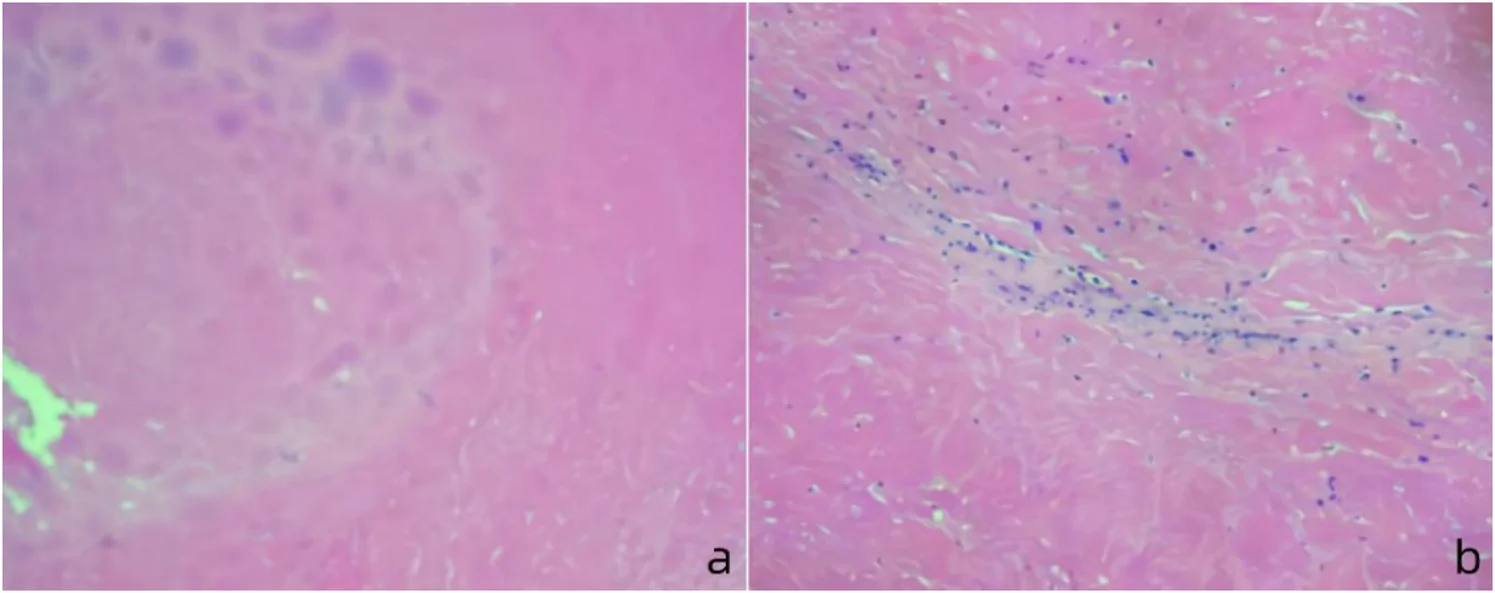
Histopathological features of rice body synovitis (hematoxylin-eosin staining, magnification ×400).

The resected synovial tissue and loose bodies were submitted for pathological examination postoperatively. The pathological findings revealed proliferative fibrous tissue in the bursa wall, extensive infiltration of lymphocytes and plasma cells, and loose bodies with a core of amorphous necrotic tissue surrounded by fibrin, consistent with rice body synovitis (Figure 5).

## 4 Treatment and prognosis

Postoperatively, the patient was administered non-steroidal anti-inflammatory drugs (NSAIDs) to alleviate pain and reduce inflammation. A staged rehabilitation program was initiated after surgery. During the early postoperative period, the shoulder was protected with a sling, and pendulum exercises and passive or assisted range-of-motion exercises were performed under guidance. Active motion and strengthening exercises were gradually introduced according to tendon healing and functional recovery. The patient's pain symptoms had significantly subsided at 1 week postoperatively, and the incision was healing well, allowing for discharge. Post-discharge follow-up was scheduled at 6 months. During follow-up, the patient's shoulder range of motion gradually returned to normal, achieving 180° abduction and 160° elevation without pain or recurrence of the mass. Ultrasound and MRI examinations revealed no abnormal masses around the shoulder joint, no synovial thickening, and no loose bodies within the joint cavity or bursa.

Given the elevated inflammatory markers and a long-standing history of RA, the patient was advised to undergo regular rheumatology follow-ups after surgery for reassessment of disease activity and optimization of DMARD therapy. Control of systemic inflammation was considered important for reducing the risk of recurrent synovitis and rice body formation.

## 5 Patient perspective

The patient reported marked pain relief and improved shoulder function after surgery. At the 6-month follow-up, he was able to perform daily activities without recurrent shoulder swelling or pain, and he expressed satisfaction with the treatment outcome.

## 6 Microbiological and differential diagnostic considerations

A structured differential diagnosis is presented in Table 3.

**Table 3:** Structured differential diagnosis.

Condition	Typical clues	Findings in this case	Reason for exclusion or lower likelihood
RA-associated rice body synovitis	Long-standing inflammatory synovitis; multiple non-calcified rice bodies; fibrin-encased necrotic cores	25-year RA history; elevated RF/anti-CCP and inflammatory markers; MRI floating lotus sign; fibrin-surrounded necrotic cores	Most consistent diagnosis
Tuberculous synovitis	Constitutional symptoms; granulomatous inflammation; positive TB tests/culture/PCR; possible bone erosion	No constitutional symptoms; no caseating granuloma; no destructive bone change	Less likely, although microbiological testing was incomplete
Synovial osteochondromatosis	Cartilaginous or ossified loose bodies; calcification; high T2 cartilage signal; chondrocytes on pathology	Radiography showed no calcification; pathology showed no cartilage matrix or chondrocytes	Not supported pathologically
Pigmented villonodular synovitis/tenosynovial giant cell tumor	Diffuse hemosiderin deposition; blooming on gradient-echo MRI; villonodular brown synovium	No diffuse hemosiderin-rich tissue; multiple white rice bodies predominated	Not supported
Lipoma arborescens/lipomatous synovitis	Frond-like fatty synovial proliferation with fat signal suppressed on fat-saturated sequences	MRI showed multiple non-fat nodules in the effusion rather than fatty villous proliferation	Not supported

## 7 Discussion

### 7.1 Pathogenesis and pathological features

The mechanism of rice body formation remains incompletely understood. The prevailing view suggests that they are closely associated with chronic inflammation of synovial tissue and microinfarction caused by ischemia and hypoxia (5, 6, 8, 15, 16). When synovial tissue remains in a prolonged inflammatory state, vascular permeability increases (5, 15, 16). Fibrinogen leaks from the bloodstream and deposits on the synovial surface. Concurrently, ischemia and hypoxia induce microinfarctions in synovial cells (6). These necrotic tissue fragments gradually slough off and become enveloped by fibrin in the synovial fluid, ultimately forming rice-grain bodies (7). Pathologically, the core of a rice-grain body consists of necrotic tissue resulting from cell death and tissue breakdown due to ischemia and hypoxia (17, 18). The periphery is tightly enveloped by collagen fibers and fibrin, a structure that helps maintain the morphology and stability of the rice-grain body (7, 15, 17).

Based on the arthroscopic findings in this patient, initial exploration revealed diffuse hyperemia and villous-nodular proliferation of the synovial tissue (Figure 3a). This represents the pathological basis for rice-grain body formation—elevated vascular permeability in the synovium under chronic inflammatory conditions allows for the persistent leakage of fibrin and inflammatory mediators, providing the material prerequisites for the deposition and remodeling into localized nodular structures. As the field of view expanded, multiple oval-shaped white nodules of varying sizes became visible within the joint cavity (Figures 3b, d, f), representing characteristic rice-grain bodies. These constitute the terminal product of synovial pathology—exuded fibrin locally aggregates within the synovium, undergoes encapsulation by synovial cells and fibrotic remodeling to form acellular dense nodules, which ultimately detach from the synovium into the joint cavity, appearing as free-floating or semi-free dense clusters. The punctate microvascular structures on the surface of individual rice-grain bodies (Figure 3e) are not intrinsic components, but rather, vascular extensions that adhere to the diseased synovium. This indicates that rice-grain bodies maintain anatomical and metabolic connections with the synovium during their early formation and explains their tendency to cluster adjacent to inflamed synovial tissue. Furthermore, the spatially distinct relationship between the rice-grain bodies and the synovium—characterized by “clear demarcation without adhesion” (Figure 3d)—reflects their pathological stage of detachment from the synovium, constituting the direct cause of free-floating space-occupying lesions within the joint cavity. These microscopic features not only constitute the core diagnostic basis for rice-grain-body synovitis but also indicate that thorough intraoperative clearance of the proliferative synovium and free rice-grain bodies is essential for controlling inflammation and improving joint function. These findings supported the rationale for removing both the proliferative synovium and the free rice bodies in this case.

Histopathological examination showed loose bodies composed of amorphous eosinophilic necrotic material surrounded by fibrinous tissue, accompanied by villous synovial proliferation and chronic inflammatory cell infiltration. No cartilage matrix, chondrocytes, caseating granulomas, or hemosiderin-rich villonodular synovium were identified. These findings supported the diagnosis of RA-associated rice body synovitis. In the setting of long-standing RA, persistent synovial inflammation may promote fibrin deposition, local ischemia, synovial microinfarction, and subsequent fibrous remodeling, ultimately contributing to rice body formation. A proposed mechanism may involve the following steps: first, chronic RA-related cytokine activation may induce endothelial injury and synovial microthrombosis; second, local ischemia may cause synovial microinfarction and tissue sloughing; third, impaired fibrinolysis may promote fibrin deposition; fourth, fibronectin cross-linking and fibrous remodeling may form a concentric shell; fifth, histiocyte aggregation may contribute to cellular encapsulation; and finally, profibrotic signaling pathways may promote collagen deposition and maturation of rice bodies.

This proposed inflammation–coagulation–fibrosis pathway may help explain rice body formation in the setting of long-standing RA. However, this mechanistic interpretation remains hypothetical and requires validation in larger studies.

### 7.2 Diagnostic value of imaging

MRI plays a central role in the diagnosis of rice body synovitis because it can demonstrate synovial proliferation, joint or bursal distension, effusion, and intra-articular or intrabursal loose bodies. In this case, the rice body-like nodules appeared low to intermediate in signal intensity on T1- and T2-weighted images and were sharply contrasted against the surrounding hyperintense effusion. After contrast administration, the thickened synovium was enhanced, whereas the nodules showed no obvious enhancement. This imaging pattern may help distinguish rice body synovitis from synovial osteochondromatosis, in which cartilaginous or ossified loose bodies may show calcification or high T2 cartilage signal (19–23).

### 7.3 Key points for differential diagnosis

In clinical practice, rice body synovitis should be differentiated from other proliferative or nodular synovial disorders to avoid misdiagnosis (9).

#### 7.3.1 Synovial osteochondromatosis

Synovial osteochondromatosis is a proliferative synovial disorder characterized by cartilaginous or ossified loose bodies. On imaging, calcification or ossification may be visible, and cartilaginous nodules may show high signal intensity on T2-weighted images. Pathologically, synovial osteochondromatosis shows cartilaginous metaplasia, chondrocytes, and cartilage matrix. In contrast, the loose bodies in this case showed no calcification, cartilage matrix, or chondrocytes, supporting the diagnosis of rice body synovitis rather than synovial osteochondromatosis (24–27).

#### 7.3.2 Pigmented villonodular synovitis

Pigmented villonodular synovitis is characterized by villonodular proliferation of the synovium (28). On MRI, the affected areas exhibit low signal intensity due to hemosiderin deposition, which is particularly evident on gradient-echo sequences (29, 30). Unlike rice body synovitis, pigmented villonodular synovitis does not typically present with numerous white rice body-like loose bodies floating within a joint effusion (31). Furthermore, joint symptoms in patients with pigmented villonodular synovitis are often persistent, unresponsive to conservative treatment, and prone to recurrence.

#### 7.3.3 Tuberculous synovitis

Tuberculous synovitis is frequently accompanied by systemic tuberculosis symptoms such as low-grade fever, night sweats, fatigue, and weight loss. Rice bodies may also occur in tuberculous synovitis, which is often associated with constitutional symptoms, granulomatous inflammation, positive microbiological tests, and, in some cases, destructive bone changes (32, 33). Laboratory findings may include a positive tuberculin skin test, with *Mycobacterium tuberculosis* potentially detected via acid-fast staining or culture. In this case, tuberculous synovitis was considered less likely because the patient had no fever, night sweats, weight loss, destructive bone changes, caseating granulomas, or purulent synovial fluid. However, because synovial fluid culture, mycobacterial culture, acid-fast staining, and PCR testing were not performed, tuberculosis and other chronic infections could not be definitively excluded. This diagnostic uncertainty is acknowledged as a limitation of this case report.

## 8 Treatment option selection

Treatment strategies for rice body synovitis should be individualized according to symptom severity, lesion burden, associated joint pathology, and the underlying systemic diseases.

Conservative treatment is primarily suitable for patients with mild symptoms and a small number of loose bodies. Immobilization is a key component of conservative management. Restricting joint movement reduces irritation of the synovium, facilitating inflammation resolution and tissue repair. Anti-inflammatory medications such as NSAIDs can alleviate inflammatory responses and relieve pain symptoms, but caution is required due to their potential adverse effects, including gastrointestinal issues. Physical therapies, such as heat application and ultrasound treatment, can promote local blood circulation, reduce swelling and pain, and improve joint function. However, conservative treatment may be insufficient for patients with severe symptoms, an extensive lesion burden, or marked mechanical restriction.

Surgical intervention is commonly considered for symptomatic rice body synovitis, particularly when numerous loose bodies, synovial proliferation, or associated structural lesions are present. Arthroscopic surgery offers advantages such as minimal trauma, rapid recovery, and fewer complications (34, 35). In this case, arthroscopy enabled clear visualization of the intra-articular and bursal lesions. Moreover, loose bodies and proliferative synovial tissue were thoroughly removed using shavers and forceps, followed by repeated irrigation of the joint cavity and bursa. This approach has been associated with improvement in pain and shoulder function (36). The patient recovered well postoperatively, with significant pain relief and gradual restoration of normal joint range of motion. No recurrence was observed during the 6-month follow-up, suggesting that arthroscopic removal of rice bodies and the proliferative synovium may be a useful diagnostic and therapeutic option for select patients. However, this finding should be interpreted cautiously because it is based on a single case with short-term follow-up. Open surgery is indicated for patients with complex conditions where arthroscopic surgery cannot completely remove the lesions. However, open surgery is more invasive, requires a longer postoperative recovery period, and carries risks of complications, such as infection and adhesions (37).

## 9 Limitations

This report has several limitations. First, it is a single case and therefore cannot establish the incidence, optimal treatment, or recurrence risk of shoulder rice body synovitis in RA. Second, follow-up was limited to 6 months; longer observation is required because RA is chronic and recurrent synovitis may develop. Third, microbiological testing was incomplete: synovial fluid culture, mycobacterial culture, acid-fast staining, and PCR testing were not performed. Although the clinical, imaging, and histological findings favored RA-associated rice body synovitis, infection cannot be excluded with the same certainty as it could with a complete microbiological panel. Fourth, the report provides limited longitudinal detail regarding RA disease activity, standardized RA activity scores, complete medication history, and DMARD adjustments before and after surgery. Future reports should include standardized RA activity scores, medication history, microbiological workup, and longer imaging follow-up.

## 10 Conclusion

RA-associated rice body synovitis should be considered in patients with long-standing RA who present with persistent shoulder swelling and MRI findings of multiple non-calcified rice body-like nodules within a hyperintense effusion. MRI, arthroscopy, and histopathology are complementary for diagnosis. Arthroscopic removal of the rice bodies and proliferative synovium, combined with repair of a concomitant rotator cuff injury, was associated with favorable short-term recovery in this case. However, this is a single case with limited follow-up, and long-term outcomes depend not only on local surgery but also on sustained control of systemic RA activity.

## Data Availability

The original contributions presented in the study are included in the article/Supplementary Material; further inquiries can be directed to the corresponding authors.
